# Telehealth Trends and Hypertension Management Among Rural and Medicaid Patients After COVID-19

**DOI:** 10.1089/tmj.2023.0628

**Published:** 2024-06-21

**Authors:** Matthew Mackwood, Oleksandra Pashchenko, Christopher Leggett, Constance Fontanet, Jonathan Skinner, Elliott Fisher

**Affiliations:** ^1^ Department of Community & Family Medicine, Geisel School of Medicine at Dartmouth, Hanover, New Hampshire, USA.; ^2^ Dartmouth-Hitchcock Medical Center, Dartmouth Health, Lebanon, New Hampshire, USA.; ^3^ The Dartmouth Institute, Geisel School of Medicine at Dartmouth, Hanover, New Hampshire, USA.; ^4^ Full Circle Health Family Medicine Residency, Boise, Idaho, USA.; ^5^ Geisel School of Medicine at Dartmouth, Hanover, New Hampshire, USA.; ^6^ Department of Economics, Dartmouth College, Lebanon, New Hampshire, USA.

**Keywords:** *equity*, *telemedicine*, *telehealth*, *medical records*, *vulnerable populations*

## Abstract

**Objective:**

*Examine the associations between rurality and low income with primary care telehealth utilization and hypertension outcomes across multiple years pre- and post-COVID-19 pandemic onset.*

**Methods:**

*We compiled electronic health record data from the mixed rural/urban Dartmouth Health system in New Hampshire, United States, on patients with pre-existing hypertension or diabetes receiving primary care in the period before (January 2018–February 2020) and after the transition period to telehealth during the COVID-19 Pandemic (October 2020–December 2022). Stratifying by rurality and Medicaid enrollment, we examined changes in synchronous (office and telehealth visits, including audio/video use) and asynchronous (patient portal or telephone message) utilization, and control of mean systolic blood pressure (SBP) <140.*

**Results:**

*Analysis included 46,520 patients, of whom 8.2% were Medicaid enrollees, 42.7% urban residents. Telehealth use rates were 12% for rural versus 6.4% for urban, and 15% for Medicaid versus 8.4% non-Medicaid. The overall postpandemic telehealth visit rate was 0.29 per patient per year. Rural patients had a larger increase in telehealth use (additional 0.21 per year, 95% CI, 0.19–0.23) compared with urban, as did Medicaid (0.32, 95% CI 0.29–0.36) compared with non-Medicaid. Among the 38,437 patients with hypertension, SBP control worsened from 83% to 79% of patients across periods. In multivariable analysis, rurality corresponded to worsened control rates compared with urban (additional 2.4% decrease, 95% CI 2.1–2.8%); Medicaid and telehealth use were not associated with worsened control.*

**Conclusions:**

*Telehealth expansion enabled a higher shift to telehealth for rural and low-income patients without impairing hypertension management.*

## Introduction

Patients in rural areas face many difficulties accessing primary care services compared with their more urban counterparts,^1–3^ with causes ranging from provider shortages^4–6^ to rural hospital closures,^7^ and overall greater distances to access care.^8,9^ In addition, patients in rural areas have a greater prevalence of conditions that benefit from regular primary care management.^10–12^ Low-income patients are likewise well recognized as having disproportionately high chronic condition burdens and issues with accessing primary care services compared with their higher income comparators, particularly in the United States.^13^

Thanks largely to the COVID-19 pandemic, telehealth is now recognized as a powerful tool that can improve access to care; the pandemic led to a rapid expansion of telehealth services in the United States,^14–16^ encouraged by public health guidance as well as reimbursement changes including reimbursement for telephone visits and payment parity for video and in-person visits.^14,16,17^ Low-income and rural-dwelling individuals stand to disproportionately benefit from improved health care access through telehealth, due to factors such as time and cost savings associated with avoiding travel, and not having to forego wages from extended time away from work for appointments.

Concerns about whether the promise of telehealth has been realized include uncertainty about whether rural and low-income populations can adequately access these modalities (the “digital divide”^18–21^), lack of recent data (only one study includes data after 2021),^14–17,22–28^ and the paucity of data on how telehealth use may have affected chronic disease management for disadvantaged populations.^29,30^ In addition, minimal data exist on telehealth in care for chronic conditions,^31,32^ especially when expanding to consider the full range of patient support tools such as the use of asynchronous interactions—messaging through patient portals for clinical advice, or telephone messages sent for provider response in addition to usual clinic visits, which are an increasing burden on primary care clinicians.^33–35^

To improve our understanding of how access to primary care has evolved, especially for less advantaged populations, our study leverages electronic health record (EHR) data from a regional health system to examine trends in primary care utilization, including in-person, telehealth, and asynchronous messages, stratifying our analyses by Medicaid enrollment and rurality. We hypothesized that Medicaid enrollees and rural patients with chronic conditions would be more likely to leverage telehealth visits and asynchronous modalities (portal messaging/unscheduled phone calls). To enhance understanding of potential trade-offs to telehealth expansion, we also examined whether telehealth users were less likely to maintain adequate blood pressure control.

## Methods

### DESIGN, SETTING, AND PARTICIPANTS

In this retrospective cohort study, to analyze longitudinal trends in primary care utilization and hypertension management before and after the COVID-19 pandemic onset, we identified adult patients aged ≥18 in 2017 with visits or problem list diagnoses before 2018 with hypertension, diabetes, or both, who were seen longitudinally with visits in both the 2017 or 2018 and the 2022 calendar years by leveraging an EHR dataset of the Dartmouth Health system.

Dartmouth Health is a multispecialty academic medical system primarily serving rural New Hampshire and parts of Vermont, with clinics across 10 discrete regions ranging from urban Concord, New Hampshire, to micropolitan Lebanon, New Hampshire, which is the site of the main academic medical center and a 1+ hour's drive from the nearest urban center. Its 35 primary care clinics provide ∼350,000 primary care visits per year to >200,000 patients.

### TIME PERIOD

Data were aggregated across two time periods: pre-pandemic, from January 2018 through the start of March 2020, and post-transition, September 2020–December 2022. September was chosen as a starting period for post-transition analyses to eliminate the “first wave” period of the pandemic, where public health mandates and rules were the predominant drivers of telehealth use and telehealth implementation (including EHR documentation of whether a visit was conducted through video or audio-only) was not yet standardized across clinic sites.

### DATASET

Data were abstracted from an EHR-based data repository by an institutional data broker, who deidentified the dataset before provision to the study team. This study was approved by the Dartmouth-Hitchcock Institutional Research Board, ID No. 02001563, before data abstraction and analysis.

### OUTCOME MEASURES

Our primary outcomes of interest were differences in differences between time periods in primary care utilization, stratified by Medicaid enrollment and rurality; and effective control of elevated blood pressure across time periods among patients with hypertension, defined as mean systolic blood pressure (SBP) <140.

Utilization rates were aggregated into synchronous and asynchronous, and then further subdivided. Synchronous visits required real-time patient and provider interaction, and were subdivided into office visits and telehealth visits. Telehealth visits were further subdivided into audio-only or video based upon provider documentation through a discrete selection in the EHR at the time of visit.

Asynchronous encounters did not entail a real-time patient–provider interaction; typically these encounters occurred through phone call to staff or a secure message on the online patient portal linked to the patient's EHR, and were either addressed by nonprovider staff or subject to response from a medical provider either directly through messaging or indirectly through office staff. Asynchronous encounters were therefore subdivided into telephone messages and patient portal messages depending on the primary means of communication.

Hypertension outcome analyses were limited only to patients with a diagnosis of hypertension (inclusive of comorbid diabetes) and included a binary outcome indicating whether a patient's mean SBP in a given time period was <140, a generally accepted target for hypertension in primary care practice.^36^ As a secondary outcome, we also examined the change in mean SBP between time periods for all patients with hypertension. We limited analysis to SBP, given its outsized clinical relevance for hypertension management compared with isolated diastolic hypertension.^36–38^

### STATISTICAL ANALYSIS

All analyses were performed in Stata version 17.0. For nonregression outputs, significance levels were calculated as follows: categorical comparisons between groups were calculated through chi-square; continuous variables reported as mean with standard deviation through ANOVA if normally distributed, and median with interquartile ranges through Wilcoxon rank-sum otherwise. Utilization rates are reported unadjusted. To calculate 95% confidence intervals for differences between groups, we calculated average marginal effects using simple least-squares regression analysis with robust standard errors, regressing the difference in visitation rate for each patient on the characteristic of interest.

Utilization analyses were stratified by rurality, defined according to Rural-Urban Commuting Area classifications,^39^ and Medicaid status, defined by any EHR-documented enrollment in Medicaid (insurance available only to low-income persons; in 2020 New Hampshire set its eligibility threshold at 138% of poverty level) at any point across the study period.

For the SBP analyses, in addition to rurality and Medicaid enrollment, we stratified by whether the patient had a telehealth visit in the post-transition period. We estimated average marginal effects through a multivariable logistic regression model for the binary SBP control variable including a variety of factors expected to be associated with successful hypertension control.

Covariates included EHR-sourced demographic and clinical data, including race, ethnicity, preferred language, age, sex, presence of diabetes, hypertension, or both, the patient's usual primary care clinic, whether the patient used the patient portal in the post-transition period, and the distance between the patient's home and clinic (based on mileage between the patient's home and primary clinic ZIP codes). Patients with a primary address Post Office (PO) box or who changed address during the pandemic period were noted and included in primary analyses, with prepandemic address used for determining rurality and distance from primary clinic. Patients with a PO box were excluded in multivariable regression analyses, which accounted for distance.

## Results

### POPULATION CHARACTERISTICS

Patient demographics are reported in *Table 1*. A total of 46,520 patients met the inclusion criteria. Average age was 64.6 (SD = 12.9); 53.1% of patients were female, 94.8% of patients were White, 98.1% non-Hispanic, 2.0% Asian, and 1.1% Black. In the sample, 98.3% preferred English for health care communication. 8.2% were on Medicaid at some point in the study period. 84.8% of patients did not move, and 11.5% had a PO box address. Rural patients comprised 57.3% of total population, with 18.2% of the study population living in small or isolated rural areas and 37.9% in large rural areas. 80.6% of patients had hypertension and were included in the relevant outcome analyses.

**Table 1.** Characteristics of Study Populations

CHARACTERISTIC	TOTAL (***N*** = 46,520)	MEDICAID ENROLLMENT		RURALITY OF PRIMARY RESIDENCE		
		NON-MEDICAID (***n*** = 42,691)	MEDICAID (***n*** = 3,829)	URBAN (***n*** = 19,887)	LARGE RURAL (***n*** = 17,628)	ISOLATED/SMALL RURAL (***n*** = 8,461)
Age, mean (SD)	64.6 (12.9)	66.3 (12.0)	54.6 (14.0)	62.9 (12.6)	66.1 (12.9)	66.0 (12.7)
Gender						
Female	53.1	52.8	63.3	52.5	53.5	53.6
Male	46.9	47.2	36.7	47.5	46.5	46.4
Race						
White	94.8	95.3	91.4	92.0	96.7	97.4
Asian	2.0	1.9	1.9	3.2	1.2	0.5
Black or African American	1.1	0.9	2.6	2.0	0.5	0.3
Other	0.5	0.4	1.2	0.5	0.4	0.4
Unknown	1.7	1.5	3.0	2.2	1.2	1.4
Ethnicity						
Non-Hispanic	98.1	98.4	94.9	96.6	99.2	99.4
Hispanic	1.9	1.6	5.1	3.4	0.8	0.6
English is preferred language	98.3	98.6	95.2	96.7	99.5	99.8
Medicaid enrollment	8.2	NA	NA	7.2	10.7	12.4
Medicare	53.1	57.5	43.6	50.7	64.8	65.6
Rurality						
Isolated or small rural	18.2	44.3	32.5	NA	NA	NA
Large rural	37.9	38.0	43.5	NA	NA	NA
Urban	42.7	17.7	24.0	NA	NA	NA
Address is PO box	11.5	11.7	13.1	3.0	13.5	28.3
Prepandemic distance from home ZIP code to primary clinic ZIP code (median, IQR)	5.3 (1.3–12)	5.3 (1.3–12)	4.0 (1.3–12.7)	4.9 (1.3–7.8)	3.8 (0–8.9)	17.9 (12.8–23.7)
Did not move over study period	84.8	86.4	75.4	83.1	87.7	82.6
Chronic condition presence before 2018						
Diabetes	73.4	74.1	74.3	81.7	67.7	66.5
Hypertension	80.6	82.1	77.5	73.6	86.3	86.0
Hypertension and diabetes	56.0	58.2	53.8	58.0	55.5	54.2

IQR, interquartile range; NA, not applicable; PO, Post Office; SD, standard deviation.

Unless otherwise noted, all numbers are the percentage of the population in each column with the specified attribute.

### OVERALL CHANGES IN UTILIZATION RATES

Overall changes in utilization rates and differences by rurality and Medicaid status are reported in *Table 2*. We examined a total of 570,875 synchronous and 1,302,443 asynchronous encounters across both time periods. Synchronous care declined by 3.3% (from 2.76 to 2.67 encounters per year between time periods), with 8.9% of all synchronous visits occurring through telehealth post-transition and a 13.1% decline in office visits. Asynchronous care increased by 25.9% (from 5.48 to 6.90)—with the majority of increase reflected in patient portal messages, which increased by 74.4% (from 1.68 to 2.93), while unscheduled telephone calls increased by 4.2% (from 3.80 to 3.96).

**Table 2.** Post-COVID-19 Pandemic Transition Changes in Primary Care Utilization Rates Among Patients with Hypertension and/or Diabetes, by Medicaid Enrollment and Rurality

PATIENT POPULATION	SYNCHRONOUS VISITS PER PATIENT PER YEAR BY TIME PERIOD^a,b^												
	TOTAL				OFFICE VISITS				TELEHEALTH VISITS				
	PREPANDEMIC	POST-TRANSITION	DIFFERENCE	DIFFERENCE FROM REFERENCE (95% CI)^d^	PREPANDEMIC	POST-TRANSITION	DIFFERENCE	DIFFERENCE FROM REFERENCE (95% CI)^d^	PREPANDEMIC	POST-TRANSITION	DIFFERENCE	DIFFERENCE FROM REFERENCE (95% CI)^d^	
Total	2.76	2.67	−0.09	NA	2.74	2.38	−0.35	NA	0.02	0.29	0.27	NA	
Medicaid status													
Non-Medicaid	2.70	2.61	−0.09	Reference	2.68	2.35	−0.33	Reference	0.02	0.26	0.24	Reference	
Medicaid	3.41	3.35	−0.06	0.03 (−0.05 to 0.12)	3.38	2.76	−0.62	−0.29 (−0.37 to −0.21)	0.02	0.59	0.57	0.32 (0.29–0.36)	
Rurality													
Urban	2.69	2.52	−0.17	Reference	2.67	2.33	−0.34	Reference	0.02	0.19	0.17	Reference	
Large rural	2.81	2.76	−0.05	0.12 (0.08–0.16)	2.80	2.42	−0.38	−0.03 (−0.07 to 0.00)	0.01	0.34	0.32	0.15 (0.14–0.17)	
Small/isolated rural	2.97	2.90	−0.07	0.11 (0.05–0.16)	2.95	2.49	−0.45	−0.11 (−0.16 to −0.06)	0.02	0.40	0.38	0.21 (0.19–0.23)	

PATIENT POPULATION	ASYNCHRONOUS ENCOUNTERS PER PATIENT PER YEAR BY TIME PERIOD^b,c^												
	TOTAL				PATIENT PORTAL MESSAGES				TELEPHONE MESSAGES				
	PREPANDEMIC	POST-TRANSITION	DIFFERENCE	DIFFERENCE FROM REFERENCE (95% CI)^d^	PREPANDEMIC	POST-TRANSITION	DIFFERENCE	DIFFERENCE FROM REFERENCE (95% CI)^d^	PREPANDEMIC	POST-TRANSITION	DIFFERENCE	DIFFERENCE FROM REFERENCE (95% CI)^d^	
Total	5.48	6.90	1.41	NA	1.68	2.93	1.25	NA	3.80	3.96	0.16	NA	
Medicaid status													
Non-Medicaid	5.31	6.63	1.33	Reference	1.70	2.90	1.20	Reference	3.61	3.74	0.13	Reference	
Medicaid	7.43	9.81	2.38	1.06 (0.79–1.32)	1.49	3.32	1.84	0.64 (0.48–0.80)	5.95	6.49	0.55	0.42 (0.23–0.61)	
Rurality													
Urban	5.18	6.24	1.06	Reference	1.68	2.70	1.02	Reference	3.51	3.55	0.04	Reference	
Large rural	5.64	7.31	1.67	0.62 (0.50–0.73)	1.48	2.90	1.42	0.40 (0.33–0.47)	4.16	4.41	0.26	0.22 (0.14–0.30)	
Small/isolated rural	6.20	7.75	1.55	0.49 (0.34–0.65)	2.21	3.65	1.44	0.42 (0.32–0.52)	3.98	4.10	0.11	0.07 (0.03–0.17)	

^a^ “Synchronous” denotes visits that occur through real-time patient–provider interaction.

^b^ Prepandemic, January 2018 through March 2020; post-transition, September 2020–December 2022.

^c^ “Asynchronous” denotes messages that did not entail a real-time patient–provider interaction, for example, between patient and nurse through phone or a secure message sent to/from provider to read later.

^d^ Estimated using average marginal effects.

All numbers reported as annual visit rate per patient per year in the population of a given row.

### DIFFERENCES IN SYNCHRONOUS AND ASYNCHRONOUS UTILIZATION

**Fig. 1.**
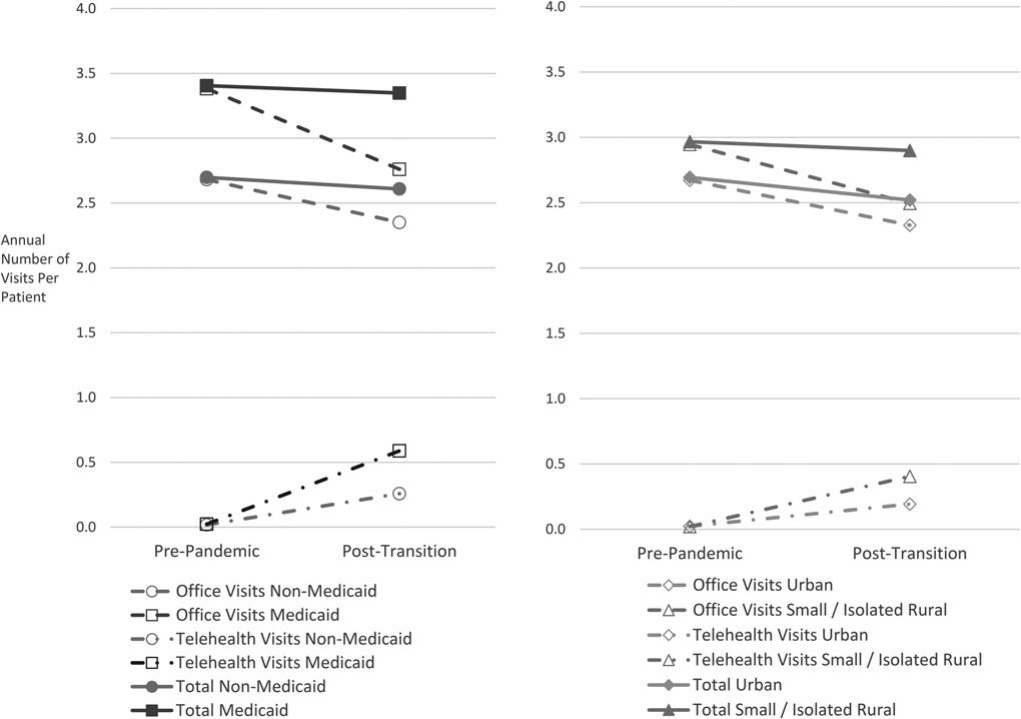
Synchronous visit rates over time by group.

**Fig. 2.**
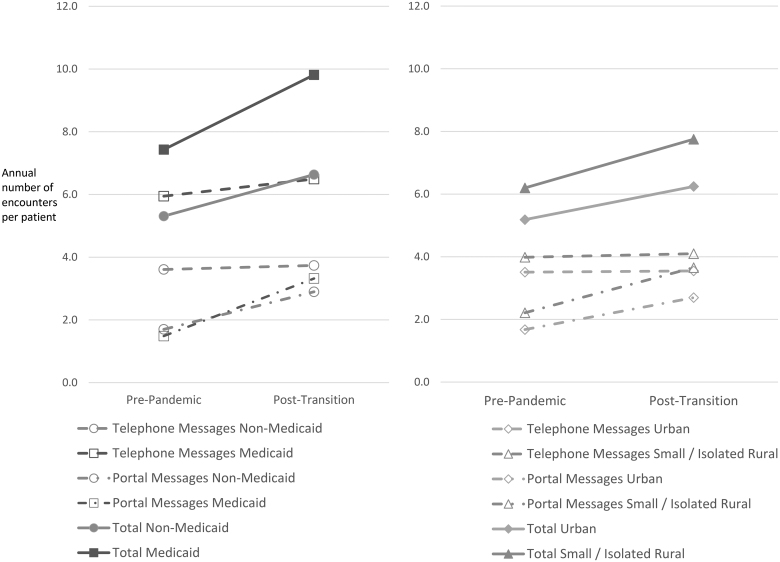
Asynchronous encounter rates over time by group.

Differences in synchronous utilization rates by group are illustrated in *Figure 1*, while *Figure 2* similarly illustrates differences in asynchronous utilization. Medicaid patients had similar rates of synchronous utilization to non-Medicaid patients in the post-transition period, and expanded asynchronous utilization more than non-Medicaid patients (1.06 additional encounters per year per patient, 95% CI 0.79–1.32). Rural patients had higher postpandemic synchronous utilization rates (difference in differences for small/isolated rural, large rural, respectively: 0.11, 95% CI 0.05–0.16, and 0.12, 95% CI 0.08–0.16) and expanded asynchronous utilization more (0.49, 95% CI 0.34–0.65, and 0.62, 95% CI 0.50–0.73) compared with urban patients.

### TELEHEALTH, AUDIO-ONLY, AND PATIENT PORTAL USE PREVALENCE AND DIFFERENCES

Telehealth-specific measures are included in *Table 3*. In the post-transition period, 30.4% of all patients used telehealth. Of these, 51.9% used audio-only for at least one of their visits. Forty-six percent of Medicaid enrollees and 38.7% of isolated/small rural patients used telehealth in the post-transition period, representing 14.9% and 12% of visits, respectively; 71.7% of all patients used patient portal messaging post-transition.

**Table 3.** Post-COVID-19 Pandemic Transition Rates of Telehealth Visits and Patient Portal Use by Medicaid Enrollment and Rurality

PATIENT POPULATION	GENERAL TELEHEALTH USE		AUDIO-ONLY TELEHEALTH USE		PATIENT PORTAL USE	
	PROPORTION OF SYNCHRONOUS ENCOUNTERS DONE VIA TELEHEALTH	PROPORTION OF ALL PATIENTS WITH ANY TELEHEALTH USE	PROPORTION OF TELEHEALTH VISITS DONE VIA AUDIO	PROPORTION OF TELEHEALTH-USING PATIENTS WHO HAD A TELE-AUDIO VISIT	PROPORTION OF ASYNCHRONOUS ENCOUNTERS DONE VIA PATIENT PORTAL MESSAGING	PROPORTION OF PATIENTS WHO USED PATIENT PORTAL MESSAGING
Overall	8.9%	30.4%	50.9%	51.9%	42.5%	71.7%
Medicaid						
No Medicaid	8.4%	29.0%	49.2%	50.4%	43.7%	72.1%
Medicaid	14.9%	46.0%	60.4%	62.6%	33.8%	65.0%
Rurality						
Urban	6.4%	23.0%	34.6%	30.1%	43.2%	72.0%
Large rural	10.3%	35.0%	57.5%	63.6%	39.7%	68.9%
Small/isolated rural	12.0%	38.7%	58.8%	60.5%	47.1%	76.9%

In terms of post-transition differences, Medicaid enrollees had more telehealth visits (0.32, 95% CI 0.29–0.36) and portal message encounters (0.64, 95% CI 0.48–0.80) compared with non-Medicaid, and were more likely to have used audio-only telehealth among telehealth users (63% vs. 50%). Rural patients used more telehealth visits (0.21, 95% CI 0.19–0.23, and 0.15, 95% CI 0.14–0.17) and patient portal encounters (0.42, 95% CI 0.32–0.52, and 0.40, 95% CI 0.33–0.47) compared with urban patients, and were twice as likely to have used audio among telehealth users (61% for small/isolated rural and 64% for large rural vs. 30% for urban).

### DIFFERENCES IN SBP CONTROL

Hypertension control outcomes are reported in *Table 4*, with additional data on blood pressure measurement and changes listed in *Table 5*. Median annual rates of encounters with recorded SBP measures were 2.2 (interquartile range = 1.3–3.6) and 2.2 (1.3–3.1) per patient for hypertensive patients in the pre- and post periods, respectively, without significant differences between groups. Mean SBP increased by 1.3 mmHg between periods.

**Table 4.** Post-COVID-19 Pandemic Transition Changes in Blood Pressure Management Among Primary Care Patients with Hypertension By Medicaid Enrollment, Rurality, and Telehealth Use

POPULATION	***N***	UNADJUSTED			ADJUSTED^a^			
		% OF PATIENTS WITH MEAN SBP <140 BY PERIOD^b^			% OF PATIENTS WITH MEAN SBP <140 BY PERIOD^b^			
		PREPANDEMIC	POST-TRANSITION	DIFFERENCE^c^	PREPANDEMIC	POST-TRANSITION	DIFFERENCE^c^	DIFFERENCE FROM REFERENCE (95% CI)^c,d^
Total	38,437	83.0	78.6	4.3	84.8	80.1	−4.6	NA
Medicaid status								
No Medicaid	35,395	82.9	78.7	4.2	84.8	80.1	−4.6	Reference
Medicaid	3,042	83.2	77.4	5.8	84.7	80.1	−4.6	−0.01 (0.22 to −0.23)
Rurality								
Urban	15,183	88.6	86.0	2.7	88.9	85.2	−3.7	Reference
Large rural	15,471	81.9	76.3	5.6	83.0	77.9	−5.1	−1.4 (−1.2 to −1.7)
Small/isolated rural	7,416	77.6	69.5	8.1	78.1	72.0	−6.1	−2.4 (−2.1 to −2.8)
Telehealth use post-transition								
Nontelehealth user	26,492	82.9	79.1	3.8	84.7	80.1	−4.6	Reference
Telehealth user	11,945	83.2	77.6	5.6	84.9	80.3	−4.6	0.03 (0.17 to −0.10)

^a^ Adjusted for age, gender, presence of comorbid diabetes, race, ethnicity, preferred language, patient's primary clinic site, distance from clinic, and patient portal message use.

^b^ Pre-pandemic, January 2018 through March 2020; post-transition, September 2020–December 2022.

^c^ Discrepancies due to rounding.

^d^ Estimated using average marginal effects.

SBP, systolic blood pressure.

All numbers reported as percentage of population in each row with systolic blood pressure ≥140.

**Table 5.** Blood Pressure Measurements Among Patients with Hypertension by Medicaid Enrollment, Rurality, and Telehealth Use

PATIENT POPULATION	***N***	ANNUAL BLOOD PRESSURE CHECK FREQUENCY, MEDIAN (IQR)				MEAN SYSTOLIC BLOOD PRESSURE (95% CI)		
		PREPANDEMIC		POST-TRANSITION		PREPANDEMIC	POST-TRANSITION	DIFFERENCE (95% CI)
Total	38,437	2.2	(1.3–3.6)	2.2	(1.3–3.1)	129.7 (129.6–129.8)	131.0 (130.9–131.1)	1.3 (1.2–1.4)
Medicaid								
No Medicaid	35,395	2.2	(1.3–3.6)	3.1	(1.8–4.5)	129.7 (129.6–129.9)	131.0 (130.9–131.1)	1.2 (1.1–1.3)
Medicaid	3,042	2.2	(1.3–3.1)	2.6	(1.3–3.9)	128.8 (128.4–129.2)	130.7 (130.2–131.1)	1.7 (1.3–2.1)
Rurality								
Urban	15,183	2.2	(1.8–3.6)	2.2	(1.3–3.1)	127.6 (127.5–127.8)	128.2 (128.0–128.4)	0.5 (0.4–0.7)
Large rural	15,471	2.2	(1.3–3.6)	2.7	(1.8–4.0)	130.7 (130.5–130.9)	132.1 (131.9–132.3)	1.4 (1.2–1.5)
Small/isolated rural	7,416	2.2	(1.3–3.1)	2.2	(1.3–3.5)	131.7 (131.4–131.9)	134.2 (134.0–134.5)	2.6 (2.3–2.8)
Telehealth use post-transition								
Nontelehealth user	26,492	2.2	(1.3–3.1)	2.2	(1.3–3.1)	129.6 (129.5–129.8)	130.9 (130.7–131.0)	1.2 (1.0–1.3)
Telehealth user	11,945	2.7	(1.8–4.0)	2.2	(1.3–3.5)	129.8 (129.6–130.0)	131.3 (131.1–131.5)	1.5 (1.3–1.7)

A total of 38,437 patients with hypertension were included for analysis of successful SBP control across time periods. Eighty-three percent had SBP <140 prepandemic, which decreased to 78.6% post-transition. Trends were similar when stratified by Medicaid enrollment and telehealth use; however, there were significant baseline disparities in mean SBP control by rurality (88.6% for urban vs. 77.6% small/isolated rural pre-pandemic; post-transition 86.0% vs. 69.5% rural).

After multivariable logistic regression adjustments, this disparity persisted within time as well as in terms of change over time, where small/isolated rural patients' proportion of SBP control under 140 declined 2.4% more than urban patients (95% CI −2.1% to −2.8%). Telehealth use and Medicaid enrollment were not associated with significant differences in SBP control rates.

### SENSITIVITY ANALYSES

Repeat analyses excluding all patients who changed address or were listed with a PO box did not substantially alter findings, nor did imputation of median distance for such patients to enable their inclusion in multivariable regression analyses. Removing the patient portal message use factor from multivariable modeling of SBP control did not meaningfully impact differences.

Neither recalculating differences in visit rates through multivariable rather than simple linear regression (incorporating the same covariates as the hypertension analysis, excepting indicators of telehealth and patient portal use; *Table 6*), nor limiting the post-transition time period to a shortened 9-month “post-pandemic” period 2 years after pandemic onset (from March 2022 through December 31, 2022) substantially altered the main findings for visit rates or hypertension control differences.

**Table 6.** Sensitivity Analysis. Adjusted Post-COVID-19 Pandemic Transition Changes in Primary Care Utilization Rates Among Primary Care Patients with Hypertension

PATIENT POPULATION	SYNCHRONOUS								
	TOTAL			OFFICE VISITS			TELEHEALTH		
	DIFFERENCE FROM REFERENCE, 95% CI^a^			DIFFERENCE FROM REFERENCE, 95% CI^a^			DIFFERENCE FROM REFERENCE, 95% CI^a^		
Medicaid status									
Non-Medicaid	Reference			Reference			Reference		
Medicaid	0.002	−0.09	0.09	−0.29	−0.37	−0.20	0.29	0.25	0.33
Rurality									
Urban	Reference			Reference			Reference		
Large rural	0.19	0.15	0.24	0.00	−0.04	0.04	0.19	0.18	0.21
Small/isolated rural	0.17	0.11	0.22	−0.08	−0.14	−0.03	0.25	0.23	0.27

PATIENT POPULATION	ASYNCHRONOUS								
	TOTAL			PATIENT PORTAL			TELEPHONE MESSAGE		
	DIFFERENCE FROM REFERENCE, 95% CI^a^			DIFFERENCE FROM REFERENCE, 95% CI^a^			DIFFERENCE FROM REFERENCE, 95% CI^a^		
Medicaid status									
Non-Medicaid	Reference			Reference			Reference		
Medicaid	0.93	0.66	1.21	0.47	0.31	0.63	0.46	0.26	0.66
Rurality									
Urban	Reference			Reference			Reference		
Large rural	0.61	0.49	0.73	0.39	0.31	0.47	0.22	0.13	0.30
Small/isolated rural	0.52	0.36	0.68	0.42	0.32	0.52	0.10	0.00	0.20

^a^ Estimated using average marginal effects.

Adjusted for age, gender, presence of comorbid diabetes, race, ethnicity, preferred language, patient's primary clinic site, and distance from clinic.

## Discussion

The rise in telehealth during the COVID-19 pandemic provided needed care to people in their homes, but there remains a question as to whether some groups with inadequate internet access or abilities might have been left behind. Using a matched sample of patients before and after the COVID-19 pandemic transition, with data through December 2022, our retrospective cohort analysis of EHR data from a multisite mixed urban–rural health care system describes disproportionately higher uptake of primary care telehealth, including higher audio-only and portal messaging use, by low-income and rural patients with diabetes or hypertension. Furthermore, in multivariable regression analysis of effective blood pressure control we found a rural–urban disparity whereby urban patients saw much higher rates of effective treatment, yet no clinically meaningful impact attributable to telehealth use.

In contrast to much of the existing literature, we found higher utilization of telehealth services for rural populations and low-income Medicaid enrollees compared with their ostensibly more digitally enabled urban or non-Medicaid counterparts. While one study with a primarily urban sample found that low-income patients had a greater increase in telehealth use compared with higher income patients,^17^ other studies examining urban–rural differences found that low-income and rural-dwelling patients had a lower uptake of telehealth services.^23–27^ Where examined, these studies reported that audio-only use was more common among rural and low-income populations, which aligns with our findings.^24,27^

Differences in our findings may be due in part to the extended, post-transition time frame, and the exclusion of immediate pandemic onset trends from March to August of 2020. Our chronic condition population may also generally have different dynamics for telehealth use compared with broader “all-comer” analyses. These findings of increased telehealth utilization in conjunction with a higher proportion of audio-only telehealth use underscore the need to continue to address known broadband access disparities for rural regions.^40,41^

Our hypertension data support the view that telehealth use *per se* is noninferior for the treatment of hypertension, echoing the work of others to date.^42,43^ We hypothesize that telehealth use helped preserve primary care contact and sustained successful chronic condition management for at least a subset of hypertensive patients. A large gap exists in examining outcomes of clinical relevance pertaining to the use of telehealth for the care of chronic conditions, and our work adds to a still-nascent understanding that telehealth use may be an effective way to provide primary care for chronic conditions beyond hypertension.^31,32,44,45^

While most studies have restricted their attention to just synchronous telehealth and in-person visits, our findings distinguish between synchronous (real-time telehealth or in-person visits) and asynchronous (unscheduled phone call or patient portal message) utilization, and suggest that further research is warranted to help shed light on the feasibility of primary care provided through asynchronous communication as a complement to, or subset of, overall primary care volumes, given asynchronous care's 2.5 times higher frequency compared with synchronous care in the postpandemic period.

The impact of asynchronous utilization on care quality is not well quantified, although the workload burden on care teams and providers from continued expansion of this typically unreimbursed work is of concern.^28,33,34^ Data on asynchronous care are particularly limited with regard to its impact on chronic conditions though generally suggests noninferiority,^46^ including for hypertension management.^47^

We found that only 30% of patients with common chronic conditions and less than half in a given population used telehealth in the aftermath of the COVID-19 transition. A topic for future research is to understand why some used telehealth while others did not. Similarly, while the pandemic provided a natural experiment to analyze observational data on telehealth use and outcomes, more robust prospective data (e.g., randomized controlled trials of telehealth vs. in-person care use) will be essential to inform care and policy decisions.

### LIMITATIONS

This study has several limitations. First, we cannot rule out the possibility that COVID-19 had a differential impact on rural versus urban areas, or on Medicaid patients compared with non-Medicare patients. However, we expect such biases to favor higher telehealth utilization by urban residents, and non-Medicaid enrollees more likely to have digital accessibility. The extended time period and post-transition nature of our analysis likely ameliorate these biases somewhat.

Second, while our sample includes a range of rural/urban geographies and socioeconomic status, the sample of Black, Hispanic, and Asian patients is small, limiting our ability to consider racial and ethnic differences in telehealth access. Third, our sample is largely limited to New Hampshire and Vermont, and thus may not be geographically representative. And fourth, the structure of the sample excluded people who changed health systems or those without persistent access to care during the pre- and postpandemic periods.

## Conclusions

In a mixed urban/rural health system, telehealth expansion, particularly audio-only telehealth, enabled rural and Medicaid populations to shift a relatively high proportion of their care to telehealth without a significantly measured adverse effect on managing high blood pressure. These data suggest a prior gap in need for more flexibility in access for these less advantaged populations, one that was met at least in part by telehealth expansion. Persistent high blood pressure management disparities for people in rural areas suggest that exploring new ways of enhancing telehealth could in the longer term promote health equity for this population.
